# Complementary feeding: Should baby be leading the way?

**DOI:** 10.1111/jhn.12988

**Published:** 2022-01-23

**Authors:** Simon C. Langley‐Evans

**Affiliations:** ^1^ School of Biosciences, University of Nottingham, Sutton Bonington Campus Loughborough UK

## COMPLEMENTARY FEEDING

The introduction of solid foods and drinks other than milk (complementary feeding) is a key developmental milestone that exerts powerful changes in terms of functional changes to the gastrointestinal tract, the immune system and metabolic processes. Diversification of the diet exposes the infant to a greater range of fatty acids and proteins, and these, as well as associated micronutrients, must be absorbed from a more varied food matrix containing complex carbohydrates, and a following meal‐based pattern of feeding. Successful complementary feeding must ensure that the requirements of the infant for nutrients are met by the dietary supply because milk is a poor source of iron, zinc, vitamin D and vitamin A, all of which are essential for the maintenance of normal growth and function. The introduction of solid foods also serves to stimulate the development of the reflexes that coordinate biting and chewing with swallowing of food. The World Health Organization has set out guidelines advising parents that all babies should be exclusively breastfed for the first 6 months of life, with no introduction of complementary foods prior to this time.^1^ At 6 months, nutritionally adequate complementary foods should be introduced, with continuation of breastfeeding to 2 years of age. The UK Department of Health generally advocates this approach, although their guidelines suggest flexibility by wording the advice as delay until “around 6 months.” It has been suggested that babies who can sit up and hold their head steady, have good hand‐eye coordination, have lost the tongue thrust reflex and are growing at a rapid rate may benefit from introduction of solids at between 4 and 6 months.

The timing of the introduction of complementary feeding is of considerable importance and so confusion among parents as a result of imprecise guidelines is unfortunate. Late introduction (after 6 months of age) puts infants at risk of malnutrition because their stores of nutrients that have carried over from fetal life will become depleted. Early introduction of solids carries a risk of choking, as well as a risk of overwhelming the capacity of the kidneys and gastrointestinal tract to handle solutes and nitrogenous waste (leading to dehydration), and also elevates the risk of gastroenteritis and allergic sensitisation. Preterm infants represent a special case and the introduction of complementary foods should be based upon their developmental stage rather than their chronological age. Early weaning is more likely in this group of infants and there may be ongoing consequences of doing so. A study of 108 preterm infants in Brazil found that use of inappropriate foods for weaning was also a problem and early introduction of ultra‐processed foods, cows milk and wheat‐based foods was associated with lower weight‐for‐age *z*‐scores at age 2 years.^2^

The introduction of complementary foods should be accomplished gradually and the full process of weaning will typically take 6 months. Throughout that time, milk should remain a key part of the diet. Feeds of breast milk or appropriate infant formula should continue, with both later being used for drinks and mixing with solid foods. The World Health Organization strongly promotes breast milk and considers that commercial follow‐on or growing‐up milks are unnecessary.^1^ Importantly, formula milks targeted at infants older than 6 months of age are not regulated in the same way as milks for younger children. This enables open marketing and, globally, their use is increasing. Although such products are able to maintain intakes of iron and other micronutrients, when infants are transitioning to solid foods, they increase the risk of waterborne infection and exposure to contaminants, and also commit families to an unnecessary cost. Their use is growing particularly quickly in areas such as the Asia‐Pacific region.^3^

## BABY‐LED WEANING

The introduction of complementary foods is a process that generally involves parents selecting foods that have either been purchased specifically for use in the weaning process (commercially produced pureed foods) or household food items that have been pureed or finely chopped at home before being offered to babies via a spoon. Over time, the child will normally start to be offered unprocessed finger foods as the next step in the transition to normal family meals. The concern has been raised that this parentally driven process can pressure children to eat rather than to experiment with textures and flavours and hence develop their own food preferences. An alternative approach termed “baby‐led weaning” is increasingly popular, particularly among families in high‐income countries, including the UK. When the baby‐led approach is adopted, infants are provided with finger foods from the initiation of weaning and self‐select from the same foods that the rest of the family are consuming.^4^ It is claimed that this enables the development of the neural pathways that control satiety and enhances motor skills. It has been argued that freeing the infant from parental pressure to eat encourages self‐regulation of appetite and will reduce risk of childhood obesity.^5^

In this issue of the *Journal of Human Nutrition and Dietetics*, two papers explore the impact of baby‐led weaning on nutrient intake in infants. A study of 36 baby‐led weaned infants compared to 60 traditionally weaned infants found that there were few differences between the groups of infants in terms of exposures to specific food groups,^6^ although nutrient intakes were different when infants were between 6 and 8 months of age. The infants in the baby‐led group had lower intakes of micronutrients (iron, iodine, zinc, vitamin B12 and vitamin D) and milk provided a greater percentage of their daily energy and saturated fat intake.^6^ The smaller study by Brown *et al*.^7^ also found that baby‐led weaning was associated with a greater proportion of energy and nutrients being delivered by breast or formula milk rather than from solid foods. This was consistent with the baby‐led approach providing a slower transition to solids. Low intakes of micronutrients as reported in the current issue of the journal^6^, ^7^ have also been reported in previous studies and may be the product of infants self‐selecting foods that are sweeter and less nutrient‐dense.^5^, ^8^ The major concern expressed about baby‐led weaning is that it may increase the risk of the infant choking because missing out the soft‐food stage of the introduction of complementary food means that large food items may be encountered before the baby has full control over mastication. However, there is no compelling evidence that choking is a particular risk.^9^

There is some support for the idea that baby‐led weaning facilitates self‐regulation of food intake in infants,^10^ although the literature is fatally compromised by bias issues. Participants in studies of complementary feeding and the baby‐led approach generally recruit well‐educated, highly motivated, predominantly breastfeeding mother–baby pairs, with strong adherence to guidance on exclusive breastfeeding for 6 months. For example, Brown *et al*.^7^ reported that 80% of their sample were educated to at least degree level and, although 69% of “traditionally” weaning mothers were breastfeeding at 6 months, 88% were doing so in the baby‐led group.^7^ Similarly, Pearce and Langley‐Evans^6^ found that baby‐led weaning mothers were more likely (86% baby led vs. 73% traditional weaning) to be breastfeeding beyond 6 months and were better educated than traditionally weaning women. To put this into context, only 75% of women in England and Wales (where these studies were conducted^6^, ^7^) even initiate breastfeeding and only 34% still breastfeed (only 1% exclusively) to 6 months. The clear sample bias issues in the literature make it difficult to dissect out any real effect of baby‐led weaning from generally strong maternal health behaviours. Although not definitively demonstrated, the general balance of opinion is that longer‐duration breastfeeding in itself protects infants from obesity.^11^ This confounds any association between baby‐led weaning and childhood obesity^5^ and almost certainly other observations that baby‐led weaning produces better growth or metabolic outcomes.

## THE NEED FOR ENHANCED EDUCATION

The first year of life is unique in that there are no other life stages where there are such specific guidelines on food and nutrition. The World Health Organization has been very clear and directive in terms of breastfeeding advice and statements about when complementary foods should be introduced.^1^ Despite this specificity that is echoed by departments of health all over the world, compliance at the population level is remarkably poor and, in most parts of the world, solid foods are often introduced earlier than 6 months of age. It has been estimated that almost 20% of US infants receive solids before 4 months, whereas, in the UK, 75% of babies may start to be weaned by 5 months.^12^, ^13^

The reasons why guidance on complementary feeding is so poorly adhered to are complex in that decisions made by individual parents on how to proceed are shaped by many factors (socio‐economic status, infant growth rate, maternal age, infant sleeping pattern), that are further modulated by local social and cultural norms. In the UK, a degree of confusion exists about the timing of weaning because the advice given to parents has been inconsistent. As a result, many parents mistrust the advice of health professionals,^14^ and prefer to make use of possibly unreliable sources to make judgements about timing and pattern of introduction of complementary foods. Many of those sources are now Internet‐based and are subject to inaccuracy, deliberate misinformation^15^ and manipulation by manufacturers of formula milks and other products aimed at very young children. Confusion, lack of understanding and family/socio‐cultural expectations drive parents to make decisions that they consider to be in the best interests of babies, but which do not sit well with the available evidence‐base. Baby‐led weaning may be one example of this. Cutting through the complex web of information and considering what may be effective drivers of appropriate weaning behaviour will require new approaches to engage parents and enhance compliance with feeding guidelines.

Sangalli et al.^16^ have demonstrated how a primary care intervention that included training for mothers on the timing of complementary feeding and the types of food that should be used could be rolled out on a large scale in Brazil. Health centres in the intervention arm of the study gave women access to counselling on how to feed babies and children within Brazilian guidelines. At 3 years of age, children in the intervention arm had lower energy, carbohydrate and fat intakes than those in the control arm. At 6 years of age, the children in the intervention arm had smaller waist circumferences and skinfold measurements.^16^ This paper shows that that appropriate exposure to professional counselling can have a beneficial impact on the nutrition and growth of young children.

It is clear that the answers to the current problems associated with complementary feeding in high‐ and middle‐income countries do not lie in asking parents to let their babies to lead the way and select their own foods. It is noteworthy that almost 60% of parents who follow baby‐led weaning use interactive media sources rather than health professionals to shape their approach to complementary feeding,^17^ which means that compliance with weaning guidelines is lower. Guidelines are already in place and have a strong evidence‐base, such that the solutions lie in promoting the guidelines, enhancing access to reliable information, improving the training of health visitors so that they are better equipped to answer questions and promote behaviour change, and ensuring that parents of young children can access resources whenever they are required. Smartphone applications have been shown to be trusted and accessible to women in need of support when breastfeeding^18^ and perhaps similar eHealth solutions that follow on from breastfeeding promotion tools might prove beneficial in guiding parents through an infant's transition from milk to solid foods. High‐quality resources are required to counteract the often poor and unreliable advice that parents extract from social media and Internet sources.
